# Transcriptional Profiling Defines Histone Acetylation as a Regulator of Gene Expression during Human-to-Mosquito Transmission of the Malaria Parasite *Plasmodium falciparum*

**DOI:** 10.3389/fcimb.2017.00320

**Published:** 2017-07-24

**Authors:** Che J. Ngwa, Meike J. Kiesow, Olga Papst, Lindsey M. Orchard, Michael Filarsky, Alina N. Rosinski, Till S. Voss, Manuel Llinás, Gabriele Pradel

**Affiliations:** ^1^Division of Cellular and Applied Infection Biology, RWTH Aachen University Aachen, Germany; ^2^Department of Biochemistry and Molecular Biology, The Pennsylvania State University University Park, PA, United States; ^3^Department of Medical Parasitology and Infection Biology, Swiss Tropical and Public Health Institute Basel, Switzerland; ^4^Department of Chemistry and Huck Center for Malaria Research, The Pennsylvania State University University Park, PA, United States

**Keywords:** histone acetylation, gene expression, malaria, gametocyte, transmission

## Abstract

Transmission of the malaria parasite *Plasmodium falciparum* from the human to the mosquito is mediated by the intraerythrocytic gametocytes, which, once taken up during a blood meal, become activated to initiate sexual reproduction. Because gametocytes are the only parasite stages able to establish an infection in the mosquito, they are crucial for spreading the tropical disease. During gametocyte maturation, different repertoires of genes are switched on and off in a well-coordinated sequence, pointing to regulatory mechanisms of gene expression. While epigenetic gene control has been studied during erythrocytic schizogony of *P. falciparum*, little is known about this process during human-to-mosquito transmission of the parasite. To unveil the potential role of histone acetylation during gene expression in gametocytes, we carried out a microarray-based transcriptome analysis on gametocytes treated with the histone deacetylase inhibitor trichostatin A (TSA). TSA-treatment impaired gametocyte maturation and lead to histone hyper-acetylation in these stages. Comparative transcriptomics identified 294 transcripts, which were more than 2-fold up-regulated during gametocytogenesis following TSA-treatment. In activated gametocytes, which were less sensitive to TSA, the transcript levels of 48 genes were increased. TSA-treatment further led to repression of ~145 genes in immature and mature gametocytes and 7 genes in activated gametocytes. Up-regulated genes are mainly associated with functions in invasion, cytoadherence, and protein export, while down-regulated genes could particularly be assigned to transcription and translation. Chromatin immunoprecipitation demonstrated a link between gene activation and histone acetylation for selected genes. Among the genes up-regulated in TSA-treated mature gametocytes was a gene encoding the ring finger (RING)-domain protein PfRNF1, a putative E3 ligase of the ubiquitin-mediated signaling pathway. Immunochemistry demonstrated PfRNF1 expression mainly in the sexual stages of *P. falciparum* with peak expression in stage II gametocytes, where the protein localized to the nucleus and cytoplasm. *Pfrnf1* promoter and coding regions associated with acetylated histones, and TSA-treatment resulted in increased PfRNF1 levels. Our combined data point to an essential role of histone acetylation for gene regulation in gametocytes, which can be exploited for malaria transmission-blocking interventions.

## Introduction

The mosquito-borne disease malaria is the most devastating infectious tropical disease in the world, causing ~200 million new cases and more than 400,000 casualties annually (World Malaria Report, WHO, 2016). Malaria is caused by intracellularly living protozoa of the genus *Plasmodium*, with *P. falciparum* being the causative agent of malaria tropica, the most severe form of malaria.

The complex life-cycle of *P. falciparum* involves an initial round of replication in the human liver and subsequent 48-h replication cycles in the red blood cells (RBCs) that are pivotal for malaria pathogenesis. The virulence of *P. falciparum* is attributed to its ability to efficiently evade the host immune response, which includes molecular escape mechanisms to avoid complement and antibody recognition with the latter particularly depending on antigenic variation (reviewed in Scherf et al., 2008; Recker et al., 2011; Dinko and Pradel, 2016; Schmidt et al., 2016).

Immune evasion of *P. falciparum* is mediated by a tightly regulated transcription program with well-coordinated sequences of gene activation and silencing caused by chromatin-mediated epigenetic regulatory mechanisms (Duraisingh et al., 2005; Freitas-Junior et al., 2005; reviewed in Duraisingh and Horn, 2016). A major part of epigenetic control involves histone post-translational modifications (PTMs). Among others, these include histone acetylation and methylation, which are mediated by specialized transferase enzymes, including histone acetyltransferases (HATs), which promote DNA accessibility, as well as histone methyl transferases (HMTs) which can either act as promotors or inhibitors of DNA accessibility, dependent on the methylation site (Lopez-Rubio et al., 2007; Sautel et al., 2007). Histone PTMs can also be reversed, e.g., via histone deacetylases (HDACs) which remove the acetyl groups and thus inhibit gene expression. The genome of *P. falciparum* encodes five plasmodial HDACs; i.e., PfHDAC1 and 3, PfHda2 and the two type III silent information regulators PfSir2A and PfSir2B (Joshi et al., 1999; Gardner et al., 2002; reviewed in Cui and Miao, 2010) and four HATs, including the previously reported MYST and PfGNC5 (Cui et al., 2007a; Miao et al., 2010). Further, the genes coding for 10 SET (Su(var)3-9-'Enhancer of zeste-Trithorax)-domain-containing HMTs, termed PfSET1-10 were identified (Cui et al., 2008; Volz et al., 2010).

To date, histone PTMs were particularly studied during the expression of virulence-associated clonally variant multigene families, like the *var* gene family, which encodes the *P. falciparum* erythrocyte membrane protein PfEMP1 (Lopez-Rubio et al., 2007, 2009; Petter et al., 2011; reviewed in Llinás et al., 2008; Cui and Miao, 2010; Duffy et al., 2014; Duraisingh and Horn, 2016). Only a single *var* gene is expressed during replication of the RBC parasites at any one time, whereas all other *var* genes remain silent. Switching *var* expression and thus PfEMP1 structure alters the antigenic type of the infected RBCs and in consequence pathogenesis of the tropical disease. The expression of *var* genes relies on epigenetic mechanisms that induce dynamic changes in the chromatin structure (reviewed in Duffy et al., 2012). Only the active *var* gene copy assumes a euchromatic state characterized by both acetylated lysine 9 and tri-methylated lysine 4 of histone 3 (H3K9ac and H3K4me3, respectively; Lopez-Rubio et al., 2007; Salcedo-Amaya et al., 2009). On the other hand, *var* gene silencing is linked to H3K9 tri-methylation (H3K9me3) and further involves Sir2A and B and the class II HDAC PfHda2 (Duraisingh et al., 2005; Freitas-Junior et al., 2005; Lopez-Rubio et al., 2009; Tonkin et al., 2009; Coleman et al., 2014).

Recent work has further unveiled epigenetic control mechanisms during gametocyte commitment, when the RBC parasites enter the sexual pathway to form gametocytes, thus enabling parasite transmission from the human to the mosquito vector (reviewed in Josling and Llinás, 2015). Gametocyte commitment, which is proposed to be triggered by environmental signals (reviewed in Kuehn and Pradel, 2010; Bennink et al., 2016), is closely linked to the plasmodial heterochromatin protein PfHP1. This regulator usually binds specifically to H3K9me3 to maintain the heterochromatin state, resulting in *var* gene silencing and suppression of gametocyte commitment (Flueck et al., 2009; Pérez-Toledo et al., 2009; Brancucci et al., 2014). Conditional depletion of PfHP1 leads to hyper-induction of gametocytes caused by the de-repression of the *ap2-g* gene, which encodes the AP2-G transcription factor, a member of the apicomplexan Apetala2/ethylene response factor (AP2/ERF) DNA-binding protein family (Balaji et al., 2005; Kafsack et al., 2014; Sinha et al., 2014). Besides PfHP1, PfHda2 appears to be involved in the silencing of *ap2-g* gene expression in non-committed parasites, probably by removing acetylated histone residues allowing for their methylation leading to the binding of PfHP1 (Coleman et al., 2014). Once the *ap2-g* gene is activated and AP2-G becomes synthesized, the protein acts as a transcriptional switch that controls gametocyte differentiation by activating the transcription of early gametocyte genes (Kafsack et al., 2014; Sinha et al., 2014; reviewed in Voss et al., 2014; Josling and Llinás, 2015).

Once gametocyte commitment is induced, a total of about 20% of plasmodial genes are specifically expressed. These are needed for gametocyte maturation, but also for preparing the parasite to rapidly adjust to the mosquito midgut environment and to undergo gametogenesis, after the gametocytes are taken up by the blood-feeding female *Anopheles* (Florens et al., 2002; Lasonder et al., 2002; Le Roch et al., 2003). A suppression subtractive hybridization study identified 126 genes that changed in expression during initiation of gametogenesis, amongst others with putative functions in signaling, cell cycle, and gene expression (Ngwa et al., 2013). However, the mechanisms underlying differential gene regulation during gametocyte maturation and gametogenesis up to date have not been investigated.

The therapeutic use of epigenetic inhibitors in treatment of cancers has been known for more than a decade, and several HDAC inhibitors like vorinostat and romidepsin have meanwhile been approved for anticancer therapy (e.g., reviewed in Schobert and Biersack, 2017; Zagni et al., 2017). Also, the antimalarial effects of inhibitors targeting HAT and HDAC enzymes have been explored in the past (e.g., Cui et al., 2007b; Andrews et al., 2008, 2009, 2012a; Chaal et al., 2010; Wheatley et al., 2010; Sumanadasa et al., 2012; Engel et al., 2015; Alves Avelar et al., 2017; Chua et al., 2017). Additional studies showed that HDAC inhibitors also exhibit gametocytocidal activities *in vitro*, indicating that the enzymes are essential for gametocytogenesis (e.g., Hansen et al., 2014; Sun et al., 2014; Trenholme et al., 2014).

To date, only a few studies have investigated the transcriptional changes in *P. falciparum* following treatment with HDAC inhibitors. An initial study reported a general deregulation of gene expression following treatment with the HDAC inhibitor trichostatin A (TSA) during the erythrocytic replication cycle with up to 60% of the genome affected (Hu et al., 2010). Follow-up experiments, though, comparing the transcription profiles in blood stage parasites following treatment with three different HDAC inhibitors, could only detect an overlap for two genes with altered expression (Andrews et al., 2012b). The effect of HDAC inhibitors on gene expression in gametocytes has hitherto not been addressed, amongst others due to the technical challenge of harvesting high numbers of pure gametocyte stages.

To unveil the potential role of histone acetylation during gene expression in gametocytes, we now have carried out a microarray-based transcriptome analysis, in which we compared the transcriptomes of gametocytes treated with TSA and of untreated gametocytes. We show that TSA-treatment results in the deregulation of 453 genes, demonstrating a crucial role of histone PMTs in preparing the parasite for human-to-mosquito transmission.

## Materials and methods

### Antibodies

Primary antibodies used in this study included: rabbit anti-(tetra)-acetyl histone H4 K5, 8, 12, 16ac (H4Kac4) (Millipore; note: according the manufacturer's material data sheet this antibody may cross-react with other acetylated histones like H2B); rabbit anti-H3K9ac (Diagenode); rabbit anti-PfHP1 (Brancucci et al., 2014); rabbit IgG antibody (Millipore); mouse/rabbit anti-Pfs230 (Ngwa et al., 2013; Simon et al., 2016), rabbit anti-Pfs25 (BEI Resources); mouse anti-Pf39 (Scholz et al., 2008); rabbit anti-HA (Sigma Aldrich); mouse anti-proteasome SU α5 (Aminake et al., 2012), and anti-PfActinI (Ngwa et al., 2013). Mouse anti-PfRNF1 was generated for this study (see below). For indirect immunofluorescence assays (IFAs), the following dilutions of the antibodies were used: anti-H4KAc4 (1:200), anti-H3K9ac (1:200), mouse/rabbit anti-Pfs230 (1:200), anti-Pfs25 (1:1,000), anti-PfRNF1 (1:20), anti-PfHP1 (1:300), and anti-proteasome SU α5 (1:50). For Western blot (WB) analysis the following dilutions were used: anti-H4Kac4 (1:1,000), anti-H3K9ac (1:1,000), anti-Pf39 (1:1,000), anti-PfActinI (1:200), anti-PfRNF1 (1:200), rabbit anti-HA (1:1,000). For chromatin immunoprecipitation (ChIP) assays, 1 μg of each antibody (anti-H4Kac4, anti-H3K9ac, anti-PfHP1, IgG) was used.

### Parasite culture

*P. falciparum* strain NF54 was used in this study. The parasites were cultivated *in vitro* in RPMI 1640 medium supplemented with 10% heat-inactivated human serum as described (Ifediba and Vanderberg, 1981) and cultures were maintained at 37°C at an atmosphere of 5% O_2_, 5% CO_2_, and 90% N_2_. Cultures were synchronized by repeated sorbitol treatment as described (Lambros and Vanderberg, 1979). To generate gametocytes, the cultures were kept at high parasitaemia and gametocytogenesis was induced following addition of lysed RBCs. As soon as stage I gametocytes started to emerge in the culture, the culture medium was supplemented with 50 mM N-acetyl glucosamine (GlcNac) for ~5 days to kill the asexual blood stages (Fivelman et al., 2007). The gametocyte culture was then maintained in normal culture medium without GlcNac until immature (stage II–IV) or mature stage V gametocytes were harvested and enriched by Percoll gradient purification (Kariuki et al., 1998). In order to obtain activated gametocytes, Percoll-enriched mature gametocytes were incubated with 100 μM xanthurenic acid (XA) for 30 min, 1 or 6 h at room temperature (RT). Giemsa-staining of purified gametocyte smears was used to confirm purity of the samples. The human erythrocyte concentrate and serum used in this study were purchased from the Department of Transfusion Medicine (University Hospital Aachen, Germany). The University Hospital Aachen Ethics commission approved all work with human blood, the donors remained anonymous, and serum samples were pooled.

### Malstat assay

To determine the antimalarial effect of the histone deacetylase inhibitor TSA (Sigma-Aldrich), a Malstat assay was performed as described previously (Aminake et al., 2011). Synchronized ring stages of *P. falciparum* strain NF54 were plated in triplicate in 96-well plates (200 μl/well) at a parasitaemia of 1% in the presence of TSA dissolved in 0.5% vol. ethanol (5 μM to 0.06 nM). Chloroquine, dissolved in double-distilled water, served as a positive control in the experiments. Incubation of parasites with ethanol alone at a concentration of 0.5% vol. was used as negative control. Parasites were cultivated *in vitro* for 72 h, resuspended, and aliquots of 20 μl were removed and added to 100 μl of the Malstat reagent in a 96-well microtiter plate. The assessment of parasite lactate dehydrogenase (pLDH) activity was obtained by adding 20 μl of a mixture of NBT (Nitro Blue Tetrazolium) and diaphorase (1:1; 1 mg/ml stock each) to the Malstat reaction, and optical densities were measured at 630 nm. Each compound was tested four times, and the IC_50_ values were calculated from variable-slope sigmoidal dose-response curves using the GraphPad Prism program version 4.

### Gametocyte toxicity test

*P. falciparum* strain NF54 parasites were grown at high parasitaemia to induce gametocyte formation. Upon appearance of stage II gametocytes, 1 ml of culture was aliquoted in triplicate in a 24-well plate in the presence of TSA at asexual blood stage IC_50_ (29 nM) and IC_90_ (0.26 μM) concentrations. Incubation of parasites with ethanol at a concentration of 0.5% vol. and chloroquine (IC_50_), dissolved in double-distilled water, were used as negative controls. The proteasome inhibitor epoxomicin (60 nM), diluted in DMSO, served as a positive control in the experiments. The gametocytes were cultivated for 10 d with daily medium replacement. For the first 2 days of cultivation, the gametocytes were treated with the inhibitors, subsequently the medium was inhibitor-free. Every second day, Giemsa-stained blood smears were prepared and the gametocytemia was evaluated by determining the numbers of gametocytes of stages II to V in a total number of 1,000 erythrocytes in triplicate. The student's *t*-test was used to determine significant differences between TSA-treated and untreated samples.

### Macrogamete and zygote development assays

Equal volumes of mature gametocyte cultures were incubated with TSA at IC_50_ or IC_90_ concentrations or with 0.5% vol. ethanol for negative control for 1 h at 37°C. Subsequently, the cultures were activated with 100 μM XA and incubated for 30 min (macrogamete development assay) or 12 h (zygote development assay) at RT. An equal volume of each sample was then coated on Teflon slides and the cells were immunolabeled with anti-Pfs25 as described below. The numbers of Pfs25-positive macrogametes or zygotes, as distinguished by their round shapes, were counted for a total number of 1,000 erythrocytes, which were visualized by differential interference contrast in triplicate, using a Leica DMLS microscope at 1,000-fold magnification. Zygotes were distinguished from macrogametes by their larger nuclei through Hoechst nuclear stain 33342 (Molecular Probes). The student's *t*-test was used to determine significant differences between TSA-treated and untreated samples.

### Indirect immunofluorescence assay

*P. falciparum* cultures (of the wild-type NF54 strain or the PfRNF1-HA-Strep-expressing transfectant line) were air-dried on glass slides and fixed for 10 min in a methanol bath at −80°C. For membrane permeabilization and blocking of non-specific binding, fixed cells were successfully incubated in 0.01% saponin/0.5% BSA/PBS and 1% vol. neutral goat serum (Sigma-Aldrich)/PBS each for 30 min at RT. For labeling of PfHP1, the air-dried samples were fixed with 4% paraformaldehyde/PBS, pH 7.4, for 10 min at RT and subsequently treated with 0.1% vol. Triton X-100/125 mM glycine (Carl Roth)/PBS at RT for 30 min, followed by blocking of non-specific binding sites with 3% BSA/PBS for 1 h. Preparations were then incubated with the primary antibody diluted in 0.01% saponin/0.5% BSA/PBS for 1.5 h each at 37°C. Binding of primary antibody was visualized by incubating the preparations with Alexa Fluor 488-conjugated goat anti-mouse or anti-rabbit IgG secondary antibody (Molecular Probes) diluted in 0.01% saponin/0.5% BSA/PBS for 1 h at 37°C. The different parasite stages were detected by double-labeling with stage-specific antibodies, i.e., polyclonal rabbit or mouse antisera directed against PfMSP1 for the detection of asexual blood stages and Pfs230 and Pfs25 for the detection of gametocytes and activated gametocytes, respectively. This was followed by incubation with Alexa Fluor 594-conjugated goat anti-rabbit or anti-mouse IgG secondary antibodies (Molecular Probes) diluted in 0.01% saponin/0.5% BSA/PBS for 1 h at 37°C. Nuclei were highlighted by treatment with Hoechst nuclear stain 33342 for 1 min at RT and cells were mounted with anti-fading solution AF2 (Citifluor Ltd) and sealed with nail varnish. In cases where double-labeling was not employed, counterstaining of erythrocytes was performed using 0.05% Evans Blue/PBS (Sigma-Aldrich) for 1 min. Digital images were taken using a Leica AF 6000 microscope and processed using Adobe Photoshop CS software.

### Western blot analysis

Percoll-enriched immature (stages II–IV) and mature (stage V) gametocytes (of the wild-type NF54 strain or the PfRNF1-HA-Strep-expressing transfectant line) were harvested as described above and erythrocytes were lysed with 0.05% saponin/PBS followed by a washing step with PBS to remove the hemoglobin. The pellets were resuspended and sonicated in NP-40 lysis buffer (50 mM Tris HCl pH 8.0, 150 mM NaCl, 1% vol. NP-40) supplemented with a protease inhibitor cocktail (Roche Diagnostics, Germany). SDS-PAGE loading buffer was then added to the lysates, heat-denatured for 10 min at 95°C, and separated via SDS-PAGE and transferred to Hybond ECL nitrocellulose membrane (Amersham Biosciences) according to the manufacturer's protocol. Membranes were blocked for non-specific binding by incubation in Tris-buffered saline containing 5% skim milk and 1% BSA, followed by incubation with the respective rabbit or mouse antibody for 2 h at RT. After washing, the membranes were incubated with an alkaline phosphatase-conjugated anti-rabbit or anti-mouse IgG secondary antibody (Sigma-Aldrich) for 1 h at RT and developed in a solution of nitroblue tetrazolium chloride (NBT) and 5-bromo-4-chloro-3-indoxyl phosphate (BCIP; Sigma-Aldrich) for 5–30 min. Scanned blots were processed using Adobe Photoshop CS software.

### Histone hyper-acetylation assay

To investigate histone hyper-acetylation caused by TSA-treatment, hyper-acetylation assays were carried out as previously described (Andrews et al., 2012b). Percoll-enriched immature (stages II–IV) and mature (stage V) gametocytes were treated with TSA at IC_90_ concentrations or with 0.5% vol. ethanol (negative control) for 1 and 6 h at 37°C, respectively. Protein lysates were generated, employed to SDS-PAGE and histone hyper-acetylation was analyzed by WB analysis using anti-H4Kac4 and anti-H3K9ac antibodies as described above. Immunoblotting with anti-Pf39 antisera was used as loading control. Histone hyper-acetylation was quantified from three to six different experiments by measuring the band intensities via the Image J programme. When immunoblotting with anti-H4Kac4 antibody, total acetylation bands were quantified. The related band intensity was normalized to Pf39 and compared with respect to untreated samples.

### Microarray analysis

Total RNA was isolated from enriched immature (stages II–IV) and mature (stage V) gametocytes and gametocytes at 1 h post-activation following treatment with TSA at IC_90_ concentrations or with 0.5% vol. ethanol (untreated control) for 1 and 6 h, respectively, using the Trizol reagent (Invitrogen) according to the manufacturer's protocol. Quality of RNA samples were assessed using a ND-1000 (NanoDrop Technologies, Thermo Scientific) and by agarose gel electrophoresis. The microarray experiments were carried out as described previously (Kafsack et al., 2012). Briefly, synthesis of first strand amino-allyl cDNA was performed using Superscript II reverse transcriptase (Invitrogen). The amino-allyl cDNA was then cleaned and concentrated using the Zymo DNA clean and concentrator-5 column (Zymo Research) followed by coupling with Cy5 dye (GE Healthcare). The reference pool consisted of a mixture RNA from asexual blood stages and gametocytes, in which synthesis of first strand amino-allyl cDNA was performed as describe above, and coupled with the Cy3 dye. Equal amounts of Cy5-labeled samples from each treatment and the Cy3-labeled reference pool were subjected to array hybridization for 17 h at 65°C using a *P. falciparum* DNA Agilent microarray chip (Agilent Technologies AMADID #037237) containing the 5,363 coding genes (Bozdech et al., 2003). The arrays were scanned using the Agilent scanner G2600D (Agilent Technologies). Normalized intensities were extracted using the Agilent Feature Extraction Software version 11.5.1.1 and uploaded to the Princeton University Microarray Database (PUMA.princeton.edu) for analysis. After background subtraction, the log2 of the (Cy5/Cy3) intensity ratio was extracted. Raw intensity data have been submitted to the NCBI Gene Expression Omnibus (GEO; http://www.ncbi.nlm.nih.gov/geo/) under accession number GSE99223. Transcript abundance of treated samples were compared to that of untreated samples. For the selection of up- and down-regulated genes, a cut-off value of greater than 2-fold for at least one of the two time-points with a consistent up- or down-regulation for both time-points was chosen. A cut-off value of greater than 2-fold for both time points was considered significant. Data were analyzed using the database PlasmoDB (http://plasmodb.org/plasmo; Aurrecoechea et al., 2009) and Microsoft Excel 2010.

### Real-time RT-PCR

To validate the microarray data, total RNA was isolated from enriched immature (stages II–IV) and mature (stage V) gametocytes following treatment with TSA at IC_90_ concentration or with 0.5% vol. ethanol (untreated control) for 1 h as described above. One microgram of each total RNA sample was used for cDNA synthesis using the SuperScript III First-Strand Synthesis System (Invitrogen), following the manufacturer's instructions. The synthesized cDNA was first tested by diagnostic PCR for asexual blood stage contamination using primers specific for the gene encoding the apical membrane antigen AMA-1 (Peterson et al., 1989; Narum and Thomas, 1994) and for gametocyte-specificity using primers specific for the gene encoding the LCCL-domain protein PfCCp2 (Pradel et al., 2004; Ngwa et al., 2013). Controls without reverse transcriptase were also used to investigate potential genomic DNA (gDNA) contamination by using *pfccp2* primers (for primer sequences, see Table S1). Primers for quantitative real time RT-PCR to confirm the up-regulation of selected genes as identified by microarray analysis were designed using the Primer 3 software (http://frodo.wi.mit.edu/primer3/) and tested on gDNA in conventional PCR to confirm primer specificity (for primer sequences, see Table S1). Real time RT-PCR measurements were performed using the Bio-Rad iQ5 Real-Time Detection System. Reactions were performed in triplicate in a total volume of 20 μl using the maxima SyBR green qPCR master mix according to manufacturer's instructions (Thermo Scientific, Germany). Controls without template and without reverse transcriptase were included in all real time RT-PCR experiments. Transcript expression levels were calculated by the 2^−ΔCt^ method (Livak and Schmittgen, 2001) using the endogenous control gene encoding the *P. falciparum* seryl tRNA-ligase (PF3D7_0717700) as reference (Salanti et al., 2003), which was confirmed not to be affected in its transcript levels by TSA-treatment.

### Chromatin immunoprecipitation-quantitative PCR

To provide evidence for a link between gene expression and histone acetylation for selected genes identified by microarray analysis, ChIP assays combined with subsequent-quantitative PCR (ChIP-qPCR) were carried as previously described (Flueck et al., 2009). Mature (stage V) gametocytes were enriched by Percoll gradient purification, treated with either 0.5% vol. ethanol (untreated) or TSA at IC_90_ concentrations for 6 h (TSA-treated) and then resuspended in RPMI 1640 medium containing human erythrocyte concentrate at 5% haematocrit. Crosslinking of gametocyte chromatin was triggered by incubation of the cultures with 1% formaldehyde (Sigma-Aldrich) for 10 min at 37°C and a termination of the reaction by addition of 0.125 M glycine diluted in doubled-distilled water. The RBCs were then lysed using 0.15% saponin/PBS and crosslinked nuclei harvested and separated from cytoplasmic proteins by the use of a 0.25 M cytoplasmic lysis buffer (20 nM Hepes, 10 mM KCl, 1 mM EDTA, 1 mM EGTA, 0.65% NP-40, 1 mM DTT, 1x protease inhibitor cocktail). The nuclei of 10^8^ mature gametocytes were then pooled and the nuclei were sheared by sonication with UPH50 (Hielscher) on ice for 40 intervals with each interval composed of 10 s sonication and 50 s resting cycles to gain fragment sizes <500 bp. A total amount of 400–450 ng chromatin was incubated under rotation with 1 μg antibody (anti-H3K9ac, anti-H4Kac4, anti-PfHP1, and rabbit IgG antibody for negative control) overnight at 4°C in the presence of 20 μl of protein A- and G-coated magnetic beads (Diagenode). After six washing steps the immunoprecipitated chromatin was eluted by adding 1% SDS and 0.1 M NaHCO_3_ as DNA elution buffer and decrosslinked at 65°C overnight. DNA purification was carried out using the PCR and Gel Clean Up Kit (Macherey-Nagel). Primers for qPCR for selected genes as identified by microarrays were designed using Primer 3 software and tested on *P. falciparum* gDNA in conventional PCR to confirm primer specificity (for primer sequences, see Table S1). Quantitative PCR measurements were performed as described above. The amount of recovered target DNA gained from untreated and TSA-treated gametocyte samples was compared to associated input DNA sample (1:10) and depicted as percentage of input for each chosen gene.

### Recombinant protein expression and production of mouse antisera

A recombinant peptide, corresponding to the N-terminal region of PfRNF1 (Figure S1A), was expressed as a maltose-binding protein-tagged fusion protein using the pMAL™c5X-vector (New England Biolabs). DNA was amplified by PCR using gene-specific primers (for primer sequences, see Table S1). Recombinant protein was expressed in *E. coli* BL21(DE3)RIL cells according to the manufacturer's protocol (Invitrogen) and isolated and affinity-purified using amylose resin according to the manufacturer's protocol (New England Biolabs). Polyclonal antisera were generated by immunization of 6-weeks old female NMRI mice (Charles River Laboratories) with subcutaneous injections of 100 μg recombinant protein emulsified in Freund's incomplete adjuvant (Sigma-Aldrich) followed by a boost after 4 weeks. Mice were anesthetized at day 10 after the boost by intraperitoneal injection of a mixture of ketamine and xylazine according to the manufacturer's protocol (Sigma-Aldrich), and immune sera were collected via heart puncture. The immune sera of three mice immunized were pooled; sera of three non-immunized mice (NMS) were used as negative control. Experiments in mice were approved by the animal welfare committee of the District Council of Cologne, Germany (ref. no. 84-02.05.30.12.097 TVA).

### Generation of a PfRNF1-HA-strep-tagged parasite line

To tag PfRNF1 with hemagglutinin (HA)-streptavidin (Strep) at the C-terminus, a 1,230 bp homologous gene fragment was amplified from *P. falciparum* NF54 gDNA using gene-specific primers (for primer sequences, see Table S1). Cloning was done using the pHAST vector (kindly provided by Alex Maier, ANU Canberra; Rug and Maier, 2013) with the help of the SacII/XhoI restriction sites. A *P. falciparum* strain NF54 culture with 5% ring stages was loaded with 100 μg of the pHAST-PfRNF1 construct in transfection buffer via electroporation (parameters: 310 V 950 μF, 13 ms; Bio-Rad gene-pulser) as described (Wirth et al., 2014). WR99210 was added to a final concentration of 2.5 nM, starting at 4 h after transfection. WR99210-resistant parasites appeared after 4 weeks. After 60–90 days of drug pressure, the respective cultures were investigated for plasmid-integration by diagnostic PCR. The gDNA of the transfected parasites was isolated using the NucleoSpin Blood Kit (Macherey-Nagel) according to the manufacturer's protocol and used as template in the diagnostic PCR to test for vector integration (for primer sequences, see Table S1; for primer location, see Figures S1B,C). Once integration was confirmed, a clonal dilution was carried out to select for single PfRNF1-HA-Strep-tagged parasite clones and one clone was used for characterization.

### Determination of protein expression of PfRNF1 following TSA-treatment

To determine if the up-regulation of PfRNF1 transcript expression following TSA-treatment corroborates with the up-regulation of protein expression, purified mature gametocytes of the *P. falciparum* wild-type NF54 strain or the PfRNF1-HA-Strep-expressing transfectant line were splitted in two equal volumes and each was pipetted into a pre-warmed 96-well plate. One part of the culture was treated with TSA at IC_90_ concentrations and the other part was treated with 0.5% vol. ethanol (untreated control). The samples were incubated for 24 h at 37°C in an atmosphere of 5% O_2_, 5% CO_2_, and 90% N_2_. Protein lysates were generated, separated via SDS-PAGE and protein levels were analyzed by WB analysis using anti-PfRNF1 antisera or anti-HA antibody as described above. Anti-Pf39 antisera was used as loading control. PfRNF1 levels were quantified for untreated and TSA-treated samples from six (for the wild-type) and two (for the transfectant) different experiments by measuring the band intensities by Image J. The related band intensities were normalized to Pf39 and compared with respect to untreated samples.

### Parasite sub-cellular fractionation

Nuclear and cytosolic fractions of *P. falciparum* strain NF54 parasites were prepared as previously described (Voss et al., 2002). Enriched immature (stages II–IV) gametocytes were treated with 0.1% saponin in PBS to lyse RBCs and washed twice with PBS. The parasite pellet was then resuspended in cold lysis buffer (20 mM Hepes, pH 7.8, 10 mM KCl, 1 mM EDTA, 1 mM DTT, 1 mM PMSF, 1% Triton X100) and incubated for 5 min on ice. The nuclei were pelleted at 2,500 g for 5 min at 4°C and the supernatant containing the cytoplasmic proteins was collected. The nuclear pellet was washed three times with lysis buffer and resuspended in twice the pellet volume of the extraction buffer (20 mM Hepes, pH 7.8, 800 mM KCl, 1 mM EDTA, 1 mM DTT, 1 mM PMSF, 1x protease inhibitor cocktail). Following incubation under rotation for 30 min at 4°C, the extract was cleared by centrifugation at 13,000 g for 30 min and 4°C. The supernatant containing the nuclear fraction was then diluted with 1 volume of dilution buffer (20 mM Hepes, pH 7.8, 1 mM EDTA, 1 mM DTT, 30% vol. glycerol). The nuclear and cytoplasmic fractions were subjected to WB using anti-PfRNF1 antisera as described above.

## Results

### TSA-treatment affects *P. falciparum* blood and sexual stage development

The effect of TSA on the blood and sexual stages of *P. falciparum* was tested *in vitro*. Malstat assays, which measure the pLDH activities demonstrated that TSA-treatment inhibited blood stage replication with a mean IC_50_ value of 29 nM and an estimated IC_90_ of 0.26 μM. Chloroquine-treatment was used for positive control in the assay and resulted in parasite growth inhibition with a mean IC_50_ value of 16 nM (Table 1).

**Table 1:** Antimalarial activities of TSA against the *P. falciparum* blood and microgamete stages.

**Assay**	**Inhibitor**	**IC_50_**	**IC_90_**
Malstat assay	TSA	29 ± 3.0 *nM*	0.26 μM
	Chloroquine	16 ± 6.0 *nM*	N/A
Exflagellation assay^a^	TSA	0.22 ± 0.040 *mM*	N/A

N/A, Not applicable;

a *published in Trenholme et al. (2014)*.

**Figure 1:**
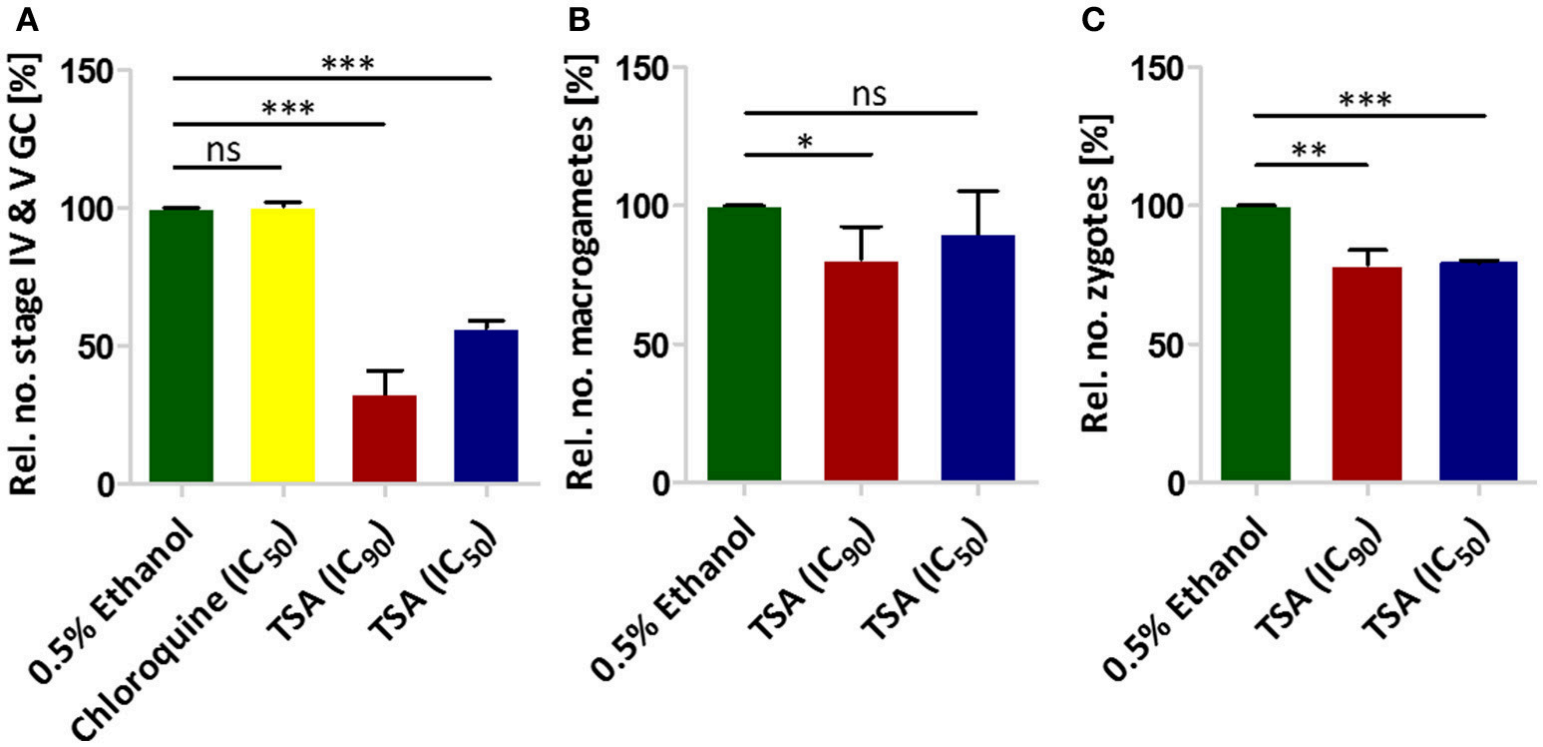
The effect of TSA on *P. falciparum* sexual stage development. **(A)** The effect of TSA on gametocyte development. TSA at IC_50_ or IC_90_ concentrations was added to stage II gametocyte cultures for 2 days. The numbers of stage IV and V gametocytes were determined in 1,000 erythrocytes at day 10 using Giemsa-stained blood smears. Epoxomicin (60 nM) was used as a positive control (not shown), while 0.5% vol. ethanol and chloroquine (16 nM) were used as negative controls (ethanol set to 100%). **(B)** The effect of TSA on macrogamete development. A mature gametocyte culture was incubated with TSA at IC_50_ or IC_90_ concentrations or 0.5% vol. ethanol for 1 h at 37°C. The culture was then activated with 100 μM XA and further cultured for 30 min at RT for macrogamete development. Macrogametes were detected by immunolabelling with anti-Pfs25 and counted in triplicate in 1,000 erythrocytes. **(C)** Effect of TSA on zygote development. The zygote development assay was performed in the same way as the macrogamete development except for the fact that after activation the cultures were incubated for 12 h at RT. Results shown (for **A–C**) are combined from three independent experiments each performed in triplicate (mean ± SD). ^*^P < 0.05; ^**^P < 0.01; ^***^P < 0.001; ns, not significant; Student's *t-*test.

We next tested the effect of TSA on gametocyte development. In this regard a culture of mainly stage II gametocytes was grown in 24-well plates in triplicate in the presence of TSA at IC_50_ and IC_90_ concentrations (as determined by Malstat assay) or in the presence of 0.5% vol. ethanol (untreated control) for 48 h. The parasites were subsequently cultured for another 8 days without inhibitor. The gametocytemia was determined every 2 days via Giemsa-stained blood smears. We showed that the numbers of stage IV and V gametocytes formed on day 10 were 43% less when the stage II gametocytes were treated with TSA at IC_50_ concentrations and 67% less when treated with IC_90_ concentrations as compared to controls (0.5% vol. ethanol and 16 nM of chloroquine) (Figure 1A). Treatment of stage II gametocytes with 60 nM epoxomicin as positive control resulted in complete elimination of stage IV and V gametocytes on day 10 (data not shown). We also investigated in detail the differentiation of gametocytes from stage II to stage V during incubation with TSA. TSA-treatment particularly affected gametocytes of stages II and III, but did not result in any delay of gametocyte maturation compared to the controls (Figure S2).

The effect of TSA on male gamete formation (termed exflagellation) has already been demonstrated for *P. falciparum* (Trenholme et al., 2014). The impairment of exflagellation by TSA is minor with IC_50_ values of 0.22 ± 0.04 mM (Table 1). To determine if TSA-treatment affects the formation of female macrogametes and zygotes, gametocyte cultures were incubated with TSA at IC_50_ and IC_90_ concentrations or with 0.5% vol. ethanol (untreated control) for 1 h at 37°C. The cultures were activated with 100 μM XA and further cultured for 30 min and 12 h to detect macrogametes and zygotes, respectively. Both stages were immunolabeled with anti-Pfs25 antibodies and counted for a total of 1,000 erythrocytes in triplicate. Comparative analyses demonstrated a slight reduction of macrogamete numbers, when these were treated with TSA at IC_90_ concentration, while the numbers of zygotes decreased by 21 and 24%, when treated with TSA at IC_50_ and IC_90_ concentrations, respectively (Figures 1B,C).

### Treatment of gametocytes with TSA causes histone hyper-acetylation

**Figure 2:**
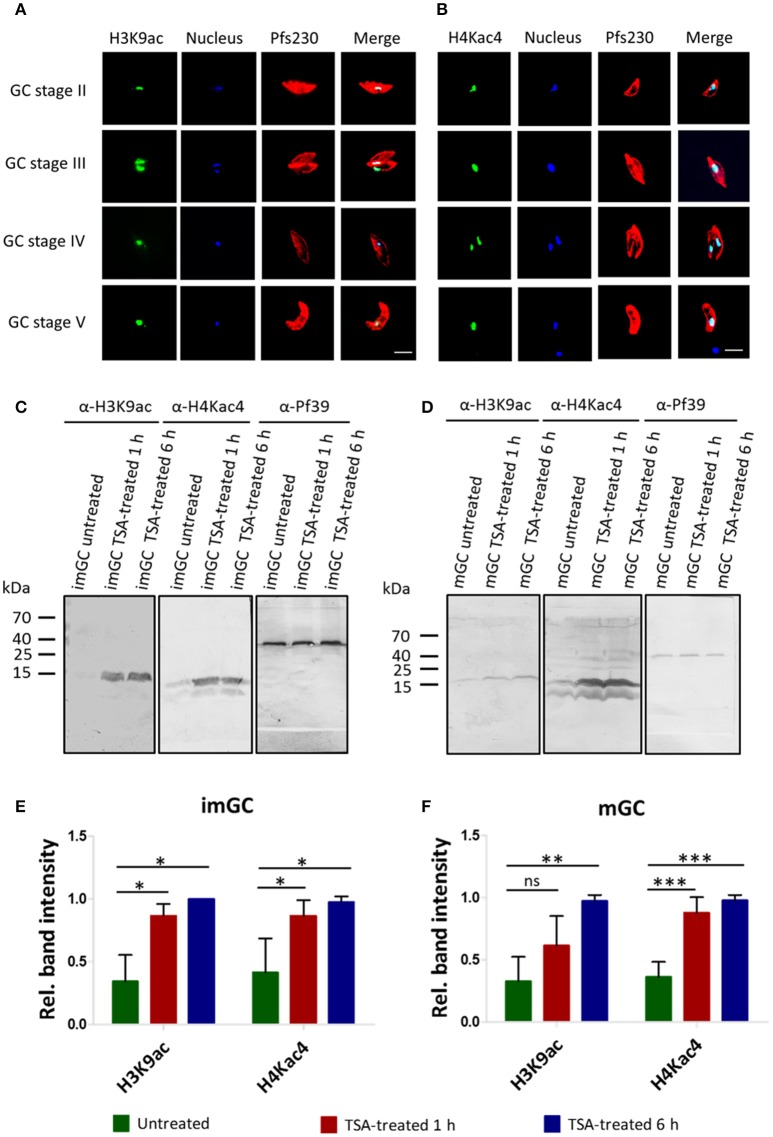
Histone acetylation and hyper-acetylation following treatment of gametocytes with TSA. **(A,B)** Presence of acetylated histones in gametocytes. Acetylated histones were detected in the different gametocyte stages (GC stage II-V) via immunolabeling using rabbit anti-H3K9ac **(A)** and anti-H4Kac4 **(B)** antibodies (green). Gametocytes were highlighted with mouse antibodies against the gametocyte marker Pfs230 (red). Nuclei were highlighted by Hoechst nuclear stain 33342 (blue). Bar, 5 μm. **(C,D)** Histone hyper-acetylation following gametocyte treatment with TSA. Protein lysates from immature gametocytes (imGC) **(C)** and mature gametocytes (mGC) **(D)** following treatment with TSA at IC_90_ concentrations or with 0.5% vol. ethanol (untreated control) for 1 and 6 h at 37°C were subjected to WB analysis using anti-H3K9ac and anti-H4Kac4 antibodies. Results shown (for **A–D**) are representative for three to six independent experiments. **(E,F)** Quantification of histone hyper-acetylation following gametocyte treatment with TSA. Lysates of imGC **(E)** and mGC **(F)** were subjected to immunoblotting as described above and histone acetylation was quantified between TSA-treated and untreated samples by measuring the band intensities via Image J for three or more different experiments; the values were normalized with the band intensities of Pf39 used as loading control (set to 1). Since the anti-H4Kac4 detected two bands in both imGC and mGC indicating that the antibody could also detect other acetylated histones, we quantified the total histone acetylation levels from both bands. ^*^P < 0.05; ^**^P < 0.01; ^***^P < 0.001; ns, not significant, Student's *t-*test. According the manufacturer's material data sheet the rabbit anti-H4Kac4 antibody can cross-react with other acetylated histones like H2B.

To assess the extent of histone acetylation during *P. falciparum* gametocyte development, two commercially available histone acetylation antibodies (anti-H3K9ac and anti-H4Kac4) were used to detect histone acetylation in gametocytes by IFA. Histone acetylation was detected throughout the nuclei of gametocyte stages II-V highlighted by Hoechst staining and immunolabeling of Pfs230, respectively (Figures 2A,B). WB analysis using the histone acetylation antibodies detected protein bands migrating at molecular weights of 15 kDa (for anti-H3K9ac) and of 11 and 13 kDa (for anti-H4Kac4). These molecular weights are in accord with the expected molecular weights of 15.4 kDa for histone H3 and 11.5 kDa for histone H4. The second protein band detected by the anti-H4Kac4 antibody might represent H2B, which has an expected molecular weight of 13.1 kDa, since the antibody also recognizes this acetylated histone as indicated in the manufacturer's material data sheet. The intensities of the protein bands increased, when the gametocytes were treated with TSA at IC_90_ concentrations, demonstrating hyper-acetylation of the histones following TSA treatment (Figures 2C,D). Quantitative WB analysis, measuring the protein band intensity of total acetylated histones showed a significant increase in their acetylation levels, when the immature and mature gametocytes were treated with TSA (Figures 2E,F).

### TSA-treatment results in the deregulation of genes during gametocyte development

We next aimed to identify genes deregulated following treatment of gametocytes with TSA. Immature, mature and activated gametocytes were treated with TSA for 1 or 6 h, total RNA was isolated, and the RNA of untreated cultures was used for comparative controls. Beforehand, the purity of the cell samples was confirmed via Giemsa smears (Figure S3A). Following cDNA synthesis, the samples were applied to a *P. falciparum* DNA Agilent microarray chip containing DNA spots corresponding to the 5,363 coding genes of nucleus, mitochondrion and apicoplast (Bozdech et al., 2003; Kafsack et al., 2012) (Table S2). Genes with transcript levels greater (i.e., up-regulated genes) or lower (i.e., down-regulated) than 2-fold compared to the untreated control (0.5% vol. ethanol) for at least one of the two time-points combined with a consistent up- or down-regulation for both time-points were used for further analysis. Changes in transcript levels greater than 2-fold for both time points compared to the control were considered significant.

**Figure 3:**
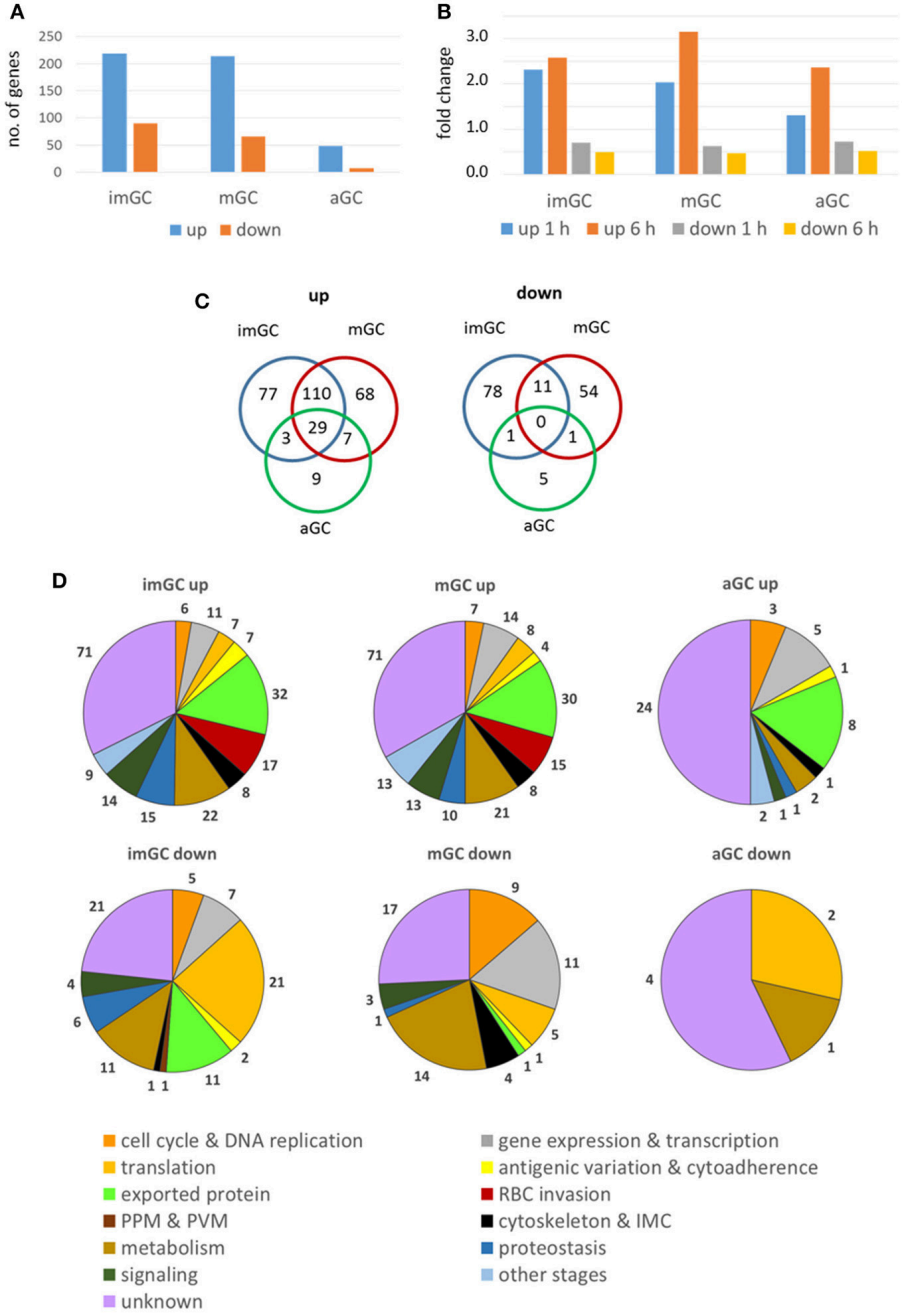
Deregulation of gene expression following treatment of gametocytes with TSA. Immature (imGC) and mature (mGC) gametocytes as well as gametocytes at 1 h post-activation (aGC) were treated with TSA at IC_90_ concentrations or with 0.5% vol. ethanol (untreated control) for 1 and 6 h, total RNA was isolated and cDNA synthesized to be employed in microarray assays. Genes with a relative expression levels greater than 2-fold for at least one of the two time-points combined with a consistent up- or down-regulation for both time-points were used for further analysis. **(A,B)** Bar charts showing total up- and down-regulated genes in imGC, mGC and aGC **(A)**, and mean fold change of deregulated genes in imGC, mGC, and aGC **(B)** at 1 or 6 h following TSA-treatment. **(C)** Venn diagram showing the overlap among deregulated genes in imGC, mGC, and aGC after TSA-treatment. **(D)** Pie chart showing the detailed number of deregulated genes in the different gametocyte samples based on the predicted function following TSA-treatment.

In immature and mature gametocytes a total of 219 and 214 genes, respectively, were identified by comparative transcript analysis that were more than 2-fold up-regulated in their expression levels, when these stages were treated with TSA. The up-regulations for 120 and 76 of these transcripts, respectively, were considered significant. Accordingly, transcripts of 90 and 66 genes were more than 2-fold down-regulated in TSA-treated immature and mature gametocytes with a significant down-regulation being observed for 8 and 11 genes, respectively. In activated gametocytes, which were less sensitive to TSA-treatment, the transcript levels of 48 genes were non-significantly increased and were decreased for 7 genes (Figure 3A, Table S3). The average up-regulation values for immature, mature and activated gametocytes were in the range of 1.3 to 3.2-fold absolute changes (immature: 1 h, 2.32; 6 h, 2.58: mature: 1 h, 2.03; 6 h, 3.16; activated: 1 h, 1,31; 6 h, 2.36), and the down-regulation values ranged between 0.7 and 0.5-fold absolute changes (immature: 1 h, 0.70; 6 h, 0.49; mature: 1 h, 0.62; 6 h, 0.47; activated: 1 h, 0.72; 6 h, 0.51) (Figure 3B). A total of 29 genes that were up-regulated in their transcript levels were shared by immature, mature and activated gametocytes, but none of the down-regulated genes were shared by the three gametocyte stages (Figure 3C).

For each of the identified genes, the microarray-based transcript data were compared with the transcriptomics data published in the database PlasmoDB. The comparison revealed that roughly 23 and 22% of the genes transcriptionally up-regulated in immature and mature gametocytes following TSA-treatment, respectively, have their peak expression in mature stage V gametocytes and in ookinetes (Figure S4; Table S3). Genes down-regulated in immature gametocytes following TSA-treatment were mainly expressed in gametocytes of stages II and V (25 and 23%, respectively), while for the mature gametocytes 58% of down-regulated genes showed their peak expression in gametocytes of stage V. In activated gametocytes, 42% of genes up-regulated after treatment with TSA were highly expressed in ookinetes, whereas all of the down-regulated genes exhibited peak expression profiles in gametocytes of stage V (Figure S4; Table S3).

The identified genes were grouped according to their predicted functions as indicated in PlasmoDB. The *in-silico* analyses demonstrated that in TSA-treated immature and mature gametocytes the up-regulated genes mainly associated with functions in gene expression and transcription and in antigenic variation and cytoadherence, or they code for exported proteins. Furthermore, in immature gametocytes, genes associated with RBC invasion and proteostasis were significantly up-regulated (Figure 3D; Figures S5A,B). The up-regulated genes mostly included ones coding for SURFINs and RIFINs, for the merozoite surface protein (MSP) family or the rhoptry neck (RON) family and for the PHIST family (Mphande et al., 2008; Proellocks et al., 2010; Beeson et al., 2016; Warncke et al., 2016). Also, genes with functions in the mosquito-specific stages were activated, when the gametocytes were treated with TSA. Down-regulated genes included ones assigned to gene expression and transcription and translation (Figure 3D). Furthermore, genes with assigned functions in metabolism and signaling as well as components of the cytoskeleton and the inner membrane complex were affected in their activation by TSA-treatment (Table S3).

In TSA-treated immature and mature gametocytes, several genes with more than 5-fold up-regulated transcription were identified, i.e., four genes in the immature gametocyte samples and 16 genes in the mature gametocyte samples (Table 2). The genes could mostly be assigned to signaling, cell cycle and DNA replication, gene expression and transcription, and proteostasis, or they included genes encoding exported proteins. Six of the genes had unknown function. One gene, encoding for the RBC invasion-related protein MSRP4 (merozoite surface protein 7-related protein 4), was identified in both immature and mature gametocytes.

**Table 2:** Genes with more than 5-fold up-regulated transcription following TSA-treatment.

**Sample**	**Plasmodb_ID**	**Gene (encoding for)**	**Group**	**Properties**	**Predicted function**	**Compartment**
Immature gametocytes	PF3D7_0113600	Surface-associated interspersed protein 1.2 (SURFIN 1.2)	Antigenic variation & cytoadherence	SICA C-terminal inner membrane domain	Cytoadherence, invasion	iRBC
	PF3D7_1334400	MSP7-like protein (MSRP4)	Invasion	1 SP, MSP7-like protein C-terminal domain	RBC invasion	Merozoite PPM
	PF3D7_0902500	Serine/Threonine protein kinase, FIKK family (FIKK9.6/TSTK9F)	Signaling	Protein kinase domain profile	Protein phosphorylation	iRBC
	PF3D7_1313100	Conserved *Plasmodium* protein	Unknown	N/A	N/A	N/A
Mature gametocytes	PF3D7_0418600	Regulator of chromosome condensation	Cell cycle & DNA replication	RCC1 domain	Regulation of chromosome condensation	Nucleus, cytoplasm
	PF3D7_0613800	Transcription factor with AP2 domain(s) (ApiAP2)	Gene expression & transcription	AP2 domain	Regulation of transcription	Nucleus
	PF3D7_0424400	Surface-associated interspersed protein 4.2 (SURFIN 4.2)	Antigenic variation & cytoadherence	N/A	Cytoadherence, invasion	iRBC
	PF3D7_1102600	*Plasmodium* exported protein (GEXP14)	Exported protein	3 TMs, export domain	N/A	iRBC
	PF3D7_1148700	*Plasmodium* exported protein (PHISTc) (GEXP12)	Exported protein	1 SP, 1 TM, RESA domain, export domain	N/A	iRBC
	PF3D7_1302300	*Plasmodium* exported protein	Exported protein	1 SP, 1 TM	N/A	iRBC
	PF3D7_1334400	MSP7-like protein (MSRP4)	Invasion	1 SP, MSP7-like protein C-terminal domain	RBC invasion	Merozoite PPM
	PF3D7_0216800	Conserved *Plasmodium* membrane protein	Metabolism	1 SP, 14 TMs, CECR6/TMEM121 family domain	Cholesterol metabolism, lipid transport	PPM
	PF3D7_0207900	Serine repeat antigen 2 (SERA2)	Proteostasis	1 SP, papain family cysteine protease profile	Proteolysis; cysteine-type peptidase activity	PV
	PF3D7_0718300	Cysteine repeat modular protein 2 (CRMP2)	Signaling	1 SP, 10 TMs, 3 GCC2 and GCC3 domains, 4 epidermal growth factor-like domains	Intracellular receptor signaling pathway, intracellular transport (protein binding)	Membrane
	PF3D7_0314800	Conserved *Plasmodium* protein	Unknown	N/A	Rosetting, cytoadherence	N/A
	PF3D7_0315600	Zinc finger protein, putative	Unknown	Zinc finger C3H1-type profile	Metal ion-binding activity	N/A
	PF3D7_0507300	Conserved *Plasmodium* protein	Unknown	1 SP	N/A	N/A
	PF3D7_0720300	conserved P*lasmodium* protein	Unknown	N/A	N/A	N/A
	PF3D7_0822700	Conserved *Plasmodium* protein	Unknown	1 SP, 3 TMs, Thrombospondin type-1 (TSP1) repeat profile	Transport (cation transmembrane transporter activity)	Membrane
	PF3D7_1350600	Conserved *Plasmodium* protein	Unknown	1 SP, 2 TMs	N/A	Membrane

*N/A, Not applicable; PPM, parasite plasma membrane; PV, parasitophorous vacuole; TM, transmembrane domain; SP, signal peptide*.

To validate the microarray array data, a total 32 genes (immature gametocytes: 15 genes; mature gametocytes: 17 genes) transcriptionally up-regulated following TSA-treatment were randomly selected. The gametocyte cultures were treated with TSA at IC_90_ concentration or 0.5% vol. ethanol (untreated control) for 1 h and total RNA was isolated. Complementary DNA was synthesized from each sample and purity was further assessed by diagnostic RT-PCR using stage specific markers. The tests confirmed the presence of *pfccp2* (gametocyte-specific) transcript in all the gametocyte samples while *ama1* (asexual blood stage-specific) transcript was absent (Figure S3B), confirming that the gametocyte samples were devoid of any asexual blood stage contamination. A test for gDNA contamination in sample preparations lacking reverse transcriptase using *pfccp2*-specific primers was negative.

**Figure 4:**
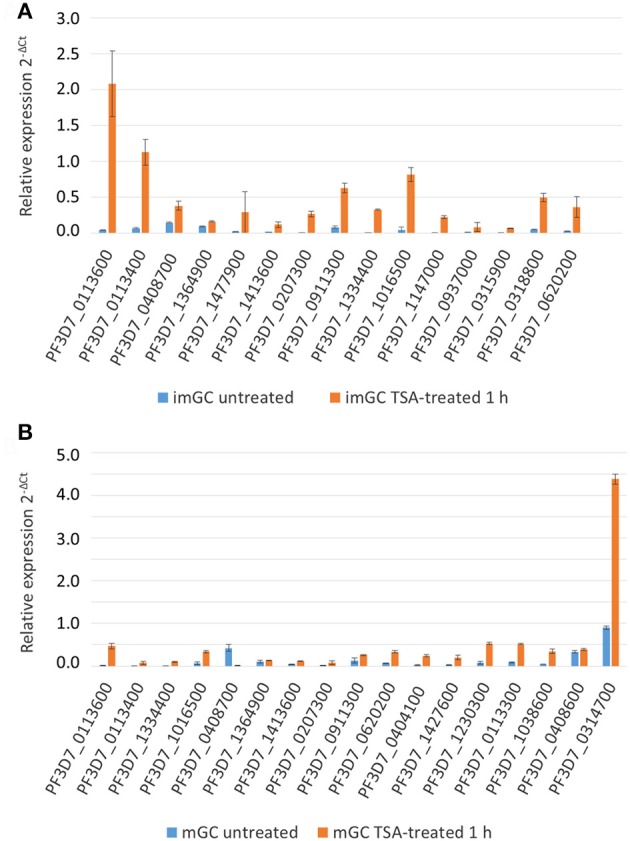
Changes in gene expression after treatment of gametocytes with TSA. Transcript analysis for 15 up-regulated immature (imGC) **(A)** and 17 up-regulated mature (mGC) **(B)** gametocyte genes as identified by microarray via real-time RT-PCR. Transcript expression levels were calculated by the 2^−ΔCt^ method; the threshold cycle number (Ct) was normalized with the Ct of the gene encoding seryl tRNA-ligase (PF3D7_0717700) as reference. Genes were considered up-regulated when the fold changes between TSA-treated and untreated sample were greater than 2-fold. Results shown are representative for two to three independent experiments.

Subsequently, the transcript expression levels of the 32 selected genes were measured via real time RT-PCR in TSA-treated and untreated samples. Transcript expression was calculated by the 2^−ΔCt^ method (Livak and Schmittgen, 2001) in which the threshold cycle number (Ct) was normalized to the Ct of the endogenous control gene encoding *P. falciparum* seryl tRNA-ligase (PF3D7_0717700) as reference gene. For TSA-treated immature gametocytes, we demonstrated a greater than 2-fold transcriptional up-regulation for 14 out of the 15 (93.3%) up-regulated genes as identified by microarray analysis (Figure 4A). Accordingly, 14 out of 17 (82.4%) of the identified up-regulated genes of the TSA-treated mature gametocyte samples had greater than 2-fold increased transcript levels compared to the untreated control (Figure 4B), which strongly validates the microarray results.

### Genes transcriptionally up-regulated following TSA-treatment associate with acetylated histones

**Figure 5:**
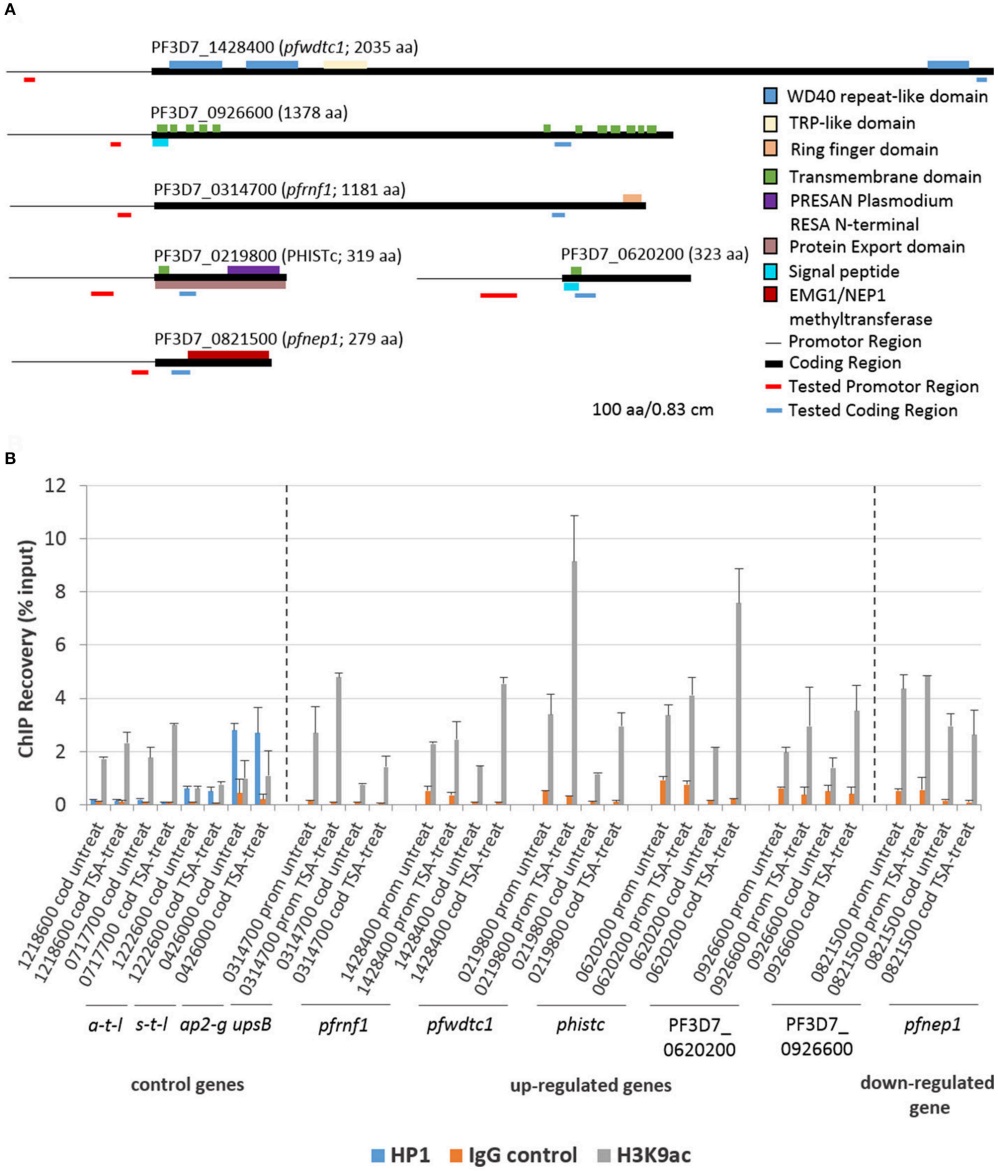
Association of genes affected by TSA-treatment with acetylated histone H3. **(A)** Predicted domain structures of the six genes deregulated in mature gametocytes by TSA-treatment. Promotor (thin black line) and coding (black box) regions as well as location of the primers specific to the promotor (red line) and to the coding region (blue line) are indicated. **(B)** ChIP-qPCR analysis of five up-regulated genes and one down-regulated gene as identified by microarray using rabbit anti-H3K9ac antibody to demonstrate potential association between the genes and acetylated histone H3. Crosslinked chromatin from mature gametocytes treated with TSA at IC_90_ concentrations or with 0.5% vol. ethanol (untreated control) for 6 h was precipitated with rabbit anti-H3K9ac antibody or with rabbit anti-PfHP1antibody and IgG as controls. The immunoprecipitated material was analyzed by qPCR to confirm specific enrichment of selected genes including *pfrnf1* (PF3D7_0314700), *pfwdtc1* (PF3D7_1428400), *phistc* (PF3D7_0219800), PF3D7_0620200, and PF3D7_0926600. As a down-regulated gene *pfnep1* (PF3D7_0821500) was analyzed. The genes encoding arginine tRNA-ligase a-t-l (PF3D7_1218600) and seryl tRNA-ligase s-t-l (PF3D7_0717700) as well as *ap2-g* (PF3D7_1222600) and the *var* gene *upsB* (PF3D7_0426000) were used as controls. Primers targeting either the coding regions (cod.) or promoter regions (prom.) were used for qPCR. The values represent the proportion of chromatin recovered from the input samples. Results shown are representative for two to three independent experiments.

ChIP-qPCR assays were employed to investigate a potential link between histone acetylation and the transcriptional up-regulation of selected genes following TSA-treatment of gametocytes. Five genes transcriptionally up-regulated in TSA-treated mature gametocytes were chosen, i.e., genes encoding a putative ring finger protein (henceforth termed *pfrnf1*, 3D7_0314700), a WD40-domain protein (*pfwdtc1*, 3D7_1428400), the exported protein PHISTc (3D7_0219800) and two unknown proteins (PF3D7_0620200, PF3D7_0926600). For control a gene transcriptionally down-regulated in TSA-treated mature gametocytes was selected, i.e., *pfnep1* (PF3D7_0821500), a gene encoding the ribosomal RNA small subunit methyl-transferase NEP1 (Figure 5A). For further controls, the housekeeping genes coding for arginine tRNA-ligase (PF3D7_1218600) and seryl tRNA-ligase (PF3D7_0717700) were chosen. Additional controls included two genes that were previously shown to associate with chromatin bound to PfHP1, i.e., *ap2-g* (3D7_1222600) and *var* gene *upsB* (PF3D7_0426000) (Brancucci et al., 2014). For each gene, primers corresponding to the promotor and coding regions were generated (for primer locations, see Figure 5A). ChIP assays were performed using anti-H3K9ac and anti-H4Kac4 antibodies, which were previously used to precipitate plasmodial chromatin (Crowley et al., 2011; Gómez-Díaz et al., 2017). For negative control, an IgG antibody from non-immunized rabbit was used in the assays. For positive control, anti-PfHP1 antibody was used to precipitate *ap2-g* and *var upsB* (Flueck et al., 2009). The ChIP recovery rates of these genes following immunoprecipitation were compared between chromatin generated from mature gametocytes treated with TSA at IC_90_ concentrations for 6 h and from untreated mature gametocytes via qPCR.

Initially, the presence of PfHP1 in the nuclei of gametocytes was demonstrated via IFA and compared to PfHP1 localization in trophozoites and schizonts. As previously described, PfHP1 as a marker for heterochromatin particularly localized to the nucleus periphery in trophozoites and schizonts (Flueck et al., 2009; Pérez-Toledo et al., 2009). Similarly, PfHP1 was predominantly found at the nucleus periphery of immature and activated gametocytes, while in mature gametocytes PfHP1 was often found in concentrated foci within the nuclei (Figure S6). Immunolabeling with NMS served as negative control and did not result in any labeling (Figure S7).

The ChIP-qPCR analysis on precipitated acetylated histones using anti-H3K9ac antibody demonstrated a higher recovery of the five genes transcriptionally up-regulated in TSA-treated mature gametocytes (Figure 5B). The increased recovery rates were detected both when primers corresponding to the promotor regions or the coding regions were used. A slightly higher recovery was also seen for the two control tRNA-ligase-encoding control genes, when the gametocytes were treated with TSA. No increased recovery rate, on the other hand, was detected, when primers corresponding to the promotor and coding regions of *pfnep1* were used in the qPCR analyses. Furthermore, the genes *ap2-g* and *var upsB* were precipitated with the anti-HP1 antibody (Figure 5B). The recovery rates for the *var* gene *upsB* were higher than the ones for *ap2-g* and neither of the recovery rates changed upon TSA-treatment of the gametocytes. Generally, similar higher recovery rates were observed for the TSA-dependent transcriptionally up-regulated genes, when the anti-H4Kac4 antibody was used for precipitation. No higher recoveries of *pfrnf1* and *pfwdtc1* were achieved, though, when the promotor regions were amplified, indicating that these promotors might associate particularly with acetylated H3 (Figure S8). Furthermore, no higher recovery for PF3D7_0620200 was detected. The overall high recovery rates observed when using anti-H4Kac4 antibody for precipitation might be due to the fact that this antibody also reacts with acetylated H2B (compare with Figures 2C,D). The combined data demonstrate an association of acetylated histones with the five selected genes that were transcriptionally up-regulated in TSA-treated gametocytes.

### Treatment of mature gametocytes with TSA results in increased PfRNF1 synthesis

**Figure 6:**
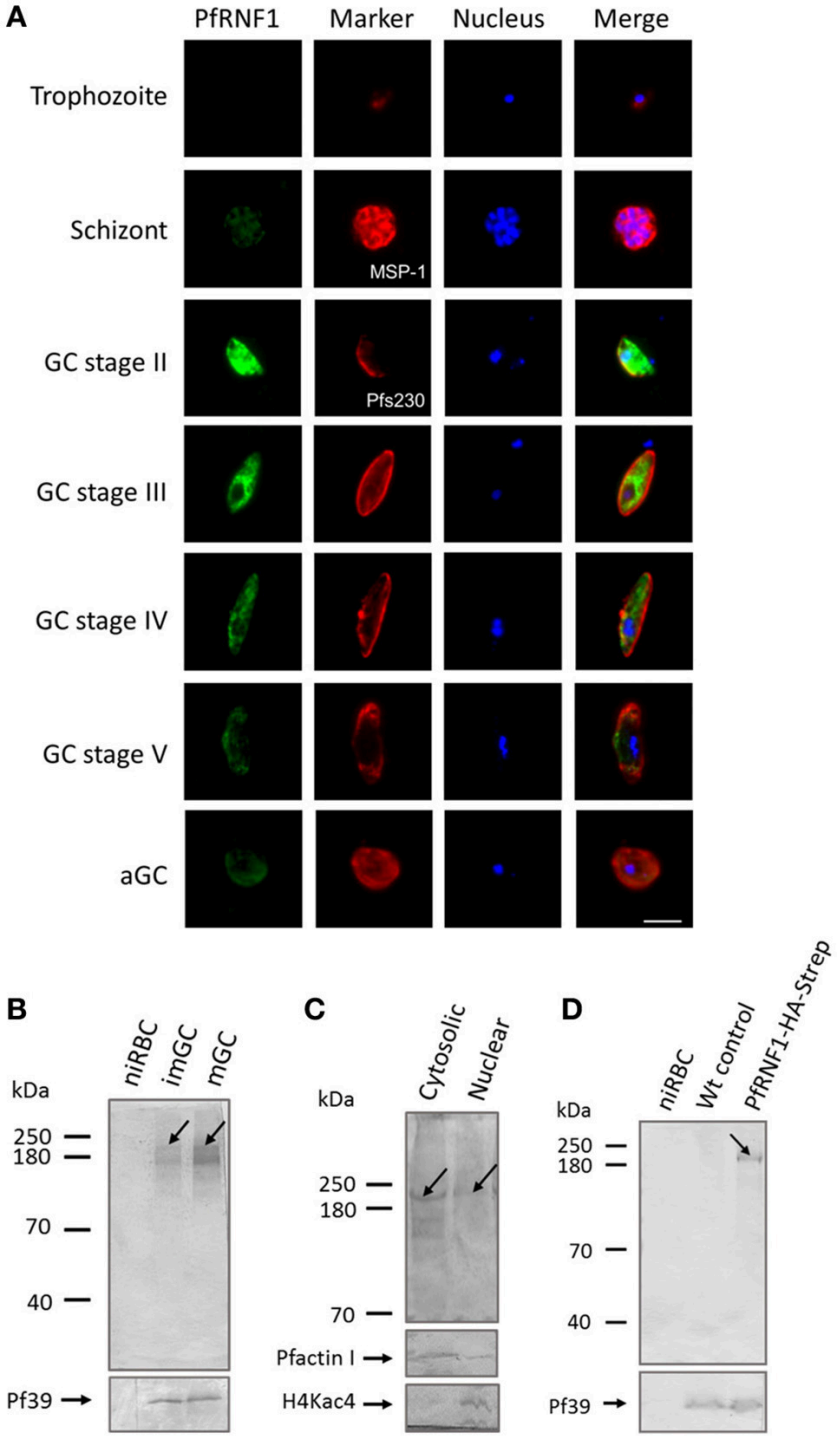
Characterization of PfRNF1. **(A)** Localization of PfRNF1 in the *P. falciparum* asexual blood and gametocyte stages. Mouse anti-PfRNF1 antisera was used to immunolabel fixed samples of trophozoites, schizonts and mature gametocytes (GC) of stages II to V as well as of activated gametocytes (aGC) at 30 min post-activation (green). Schizonts were visualized by labeling with rabbit anti-MSP-1 antibody and gametocytes were visualized by rabbit anti-Pfs230 antisera (red); nuclei were highlighted by Hoechst nuclear stain 33342 (blue). Bar, 5 μm. Results shown are representative for four independent experiments. **(B)** Expression of PfRNF1 in gametocyte lysates of immature (imGC) and mature (mGC) gametocytes were immunoblotted with anti-PfRNF1 antibody and detected two protein bands of approximate molecular weights of 200 kDa (arrow) and 140 kDa. Lysates of non-infected red blood cells (niRBCs) were used for negative control. Immunoblotting with mouse anti-Pf39 antisera served as loading control. **(C)** Sub-cellular localization of PfRNF1. Cytosolic and nuclear fractions of enriched immature gametocytes were subjected to WB using anti-PfRNF1 antisera and detected a 200-kDa band (arrow) in both fractions. Mouse antibodies against PfActinI (41 kDa) and rabbit antibodies against H4Kac4 detecting acetylated histone H4 (~11 kDa) were used as fraction controls. **(D)** Detection of PfRNF1-HA-Strep in gametocytes of parasite line PfRNF1-HA-Strep. Lysates of PfRNF1-HA-Strep immature gametocytes were immunoblotted with rabbit anti-HA antibody and detected a protein band of 200 kDa (arrow). Lysates of non-infected red blood cells (niRBCs) as well as wild-type (Wt) gametocytes were used as negative control. Results shown (for **A–D**) are representative for three to four independent experiments.

In a final set of experiments, we aimed to determine, if up-regulation of transcript expression following TSA-treatment also corroborates with increased protein synthesis. We selected one gene, *pfrnf1*, whose transcript level was up-regulated in mature gametocytes following TSA-treatment, for further investigations. *In-silico* analyses disclose the ring finger-domain protein PfRNF1 as a putative E3-ligase (Ponts et al., 2008). A recombinant protein corresponding to the N-terminal region of PfRNF1 was bacterially expressed and used to generate antisera against PfRNF1 in mice. IFA were performed in the different asexual and sexual blood stages of *P. falciparum*. Asexual blood stage parasites were highlighted by MSP-1 labeling; gametocytes and activated gametocytes were highlighted by Pfs230 immunolabeling. The IFA revealed a prominent expression of PfRNF1 in immature gametocytes with peak expression in stage II gametocytes. Here, the protein localized to the gametocyte cytoplasm and nucleus (Figure 6A). PfRNF1 was also detected at low levels in mature gametocytes and in gametocytes 30 min post-activation as well as in schizonts.

Expression of PfRNF1, which has a calculated molecular weight of 135 kDa, was then investigated in immature and mature gametocytes via WB analysis. A predominant protein band was detected in both, immature and mature gametocyte lysates, when immunoblotted with anti-PfRNF1 antisera, which was migrating at a molecular weight of roughly 200 kDa. An additional protein band at ~140 kDa was also observed (Figure 6B). No protein bands were present in lysates of the non-infected RBC control. For loading control, immunoblotting with anti-Pf39 antisera, directed against the endoplasmic reticulum-specific protein Pf39 (Templeton et al., 1997), was performed, and Pf39 was detected in all parasite lysates. We also investigated the sub-cellular localization of PfRNF1 in gametocytes via WB, using cytosolic and nuclear fractions of mixed gametocyte cultures. WB revealed the presence of PfRNF1 as a 200-kDa protein in both fractions (Figure 6C). Antibodies against PfActin1, which is predominantly present in the cytoplasm and to a minor level in the nuclei were used as a fraction control. The purity of the fractions was further confirmed by immunoblotting with anti-H4Kac4 antibody, which labeled histones in the nuclear fraction, but which did not result in any protein band in the cytoplasmic fraction (Figure 6C).

To validate the protein expression data, we generated a *P. falciparum* transfectant line, which expresses PfRNF1 C-terminally tagged with HA-Strep (Figure S1B). Successful integration was confirmed by diagnostic PCR (Figure S1C). Subsequent WB analyses, using rabbit anti-HA antibody, detected a protein band with an approximate molecular weight of 200 kDa in lysates of immature gametocytes from the transfectant line, but not from wild-type parasites used as a negative control (Figure 6D). Further, no protein band was detected in lysates of non-infected RBCs. The combined WB data indicate that PfRNF1 migrates at a higher molecular weight than expected, which might be caused by PTMs. When the PfRNF1-HA-Strep-expressing parasite line was used in IFA and immunolabeled with anti-HA antibody, a similar protein expression pattern was observed as has been described above (Figure S9).

**Figure 7:**
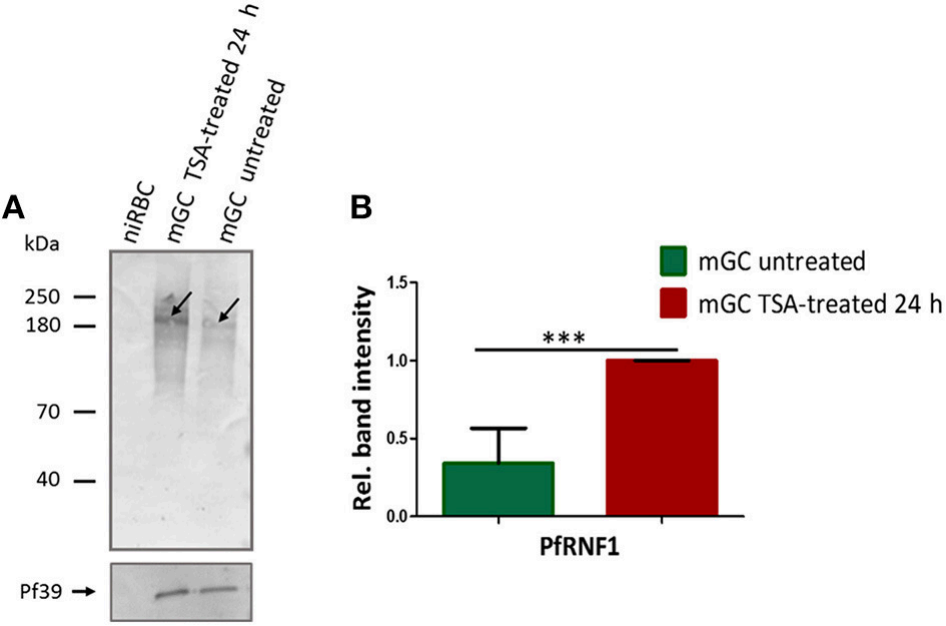
Up-regulation of PfRNF1 levels in mature gametocytes following TSA-treatment. **(A)** Lysates from mature gametocytes (mGC) following treatment with TSA at IC_90_ concentrations or 0.5% vol. ethanol (untreated control) for 24 h were subjected to WB using mouse anti-PfRNF1 antisera and detected a 200 kDa band (arrow). Immunoblotting of lysates of non-infected red blood cells (niRBC) was used for negative control; immunoblotting with mouse anti-Pf39 antisera served as loading control. **(B)** Quantification of PfRNF1 protein expression following TSA-treatment of mature gametocytes. Lysates of mature gametocytes were subjected to immunoblotting as described above and PfRNF1 levels were quantified between TSA-treated and untreated samples by measuring the band intensities via Image J for six different experiments; the values were normalized with the band intensities of Pf39 used as loading control (set to 1). Results shown (for **A,B**) are representative for six independent experiments. ^***^P < 0.001, Student's *t-*test.

Finally, we assessed whether treatment of mature gametocytes with TSA results in up-regulation of PfRNF1 on the protein level. In this regard, mature gametocytes, both of the wild-type strain and the PfRNF1-HA-Strep-expressing transfectant line, were treated with TSA at IC_90_ concentration or 0.5% vol. ethanol (untreated control) for 24 h, lysates were generated and PfRNF1 was detected by WB analysis using the anti-PfRNF1 antisera. Immunoblotting revealed increased PfRNF1 levels in lysates of TSA-treated gametocytes compared to the untreated control for both wild-type and transfectant (Figure 7A, Figure S10A). Quantitative WB analysis showed a significant up-regulation of PfRNF1 following TSA-treatment as compared to the untreated control (Figure 7B, Figure S10B).

## Discussion

Histone PTMs are emerging as major regulatory mechanisms thought to modulate gene expression in eukaryotes. While these mechanisms have been extensively studied during the erythrocytic replication of *P. falciparum*, little is known about this process during parasite human-to-mosquito transmission. In this study, we aimed to determine the role of histone acetylation and deacetylation in the control of gene expression in *P. falciparum* gametocytes during their development and transmission to the mosquito. To achieve our goal, we used the chemical loss-of-function approach using the HDAC inhibitor TSA (inhibitor of HDACs I and II) on the *P. falciparum* gametocyte-producing NF54 strain. Treatment with TSA results in a mean IC_50_ value of 29 nM. The killing of the *P. falciparum* asexual blood stages by TSA with IC_50_ values in nM ranges have already been reported before for the chloroquine-sensitive 3D7 (8 nM) and chloroquine resistant strain DD2 (11 nM) (Andrews et al., 2008).

In order to determine the effect of TSA on gametocyte development and transmission to the mosquito, we treated stage II gametocytes with the HDAC inhibitor and followed gametocyte development. We show that gametocyte development was strongly affected, and here stage II and III gametocytes appeared to be more vulnerable to TSA treatment then stage IV and V gametocytes. On the other hand, TSA only moderately effected macrogamete and zygote development. In a previous study, it was also reported that microgametes were only moderately affected following treatment with a variety of HDAC inhibitors (Trenholme et al., 2014). The fact that TSA has strong gametocytocidal activities, but only exerts minor effects on gametes and zygotes indicates that histone acetylation-mediated gene regulation is important during gametocytogenesis, but not during the early phase of midgut-stage development. This is in accord with findings in *P. falciparum* and *P. berghei* that transcript required for the midgut stage formation is synthesized and stored in female gametocytes, where it is translationally repressed by binding to regulatory ribonucleoprotein complexes, like the RNA helicase DOZI (development of zygote inhibited) and the Sm-like factor CITH (homolog of worm CAR-I and fly Trailer Hitch), as identified in *P. berghei*, or the Pumilio/FBF (Puf) family RNA-binding protein Puf2, as was shown for *P. falciparum* (Mair et al., 2006, 2010; Miao et al., 2013; Guerreiro et al., 2014). A recent analysis integrating transcriptome and proteome data revealed 512 highly expressed transcripts in *P. falciparum* female gametocytes without corresponding protein expression, indicating large scale translational repression (Lasonder et al., 2016). Repression of the stored transcript is released and translation is initiated, once the gametocytes become activated in the mosquito midgut.

To confirm histone acetylation in gametocytes, we used two commercially available histone acetylation antibodies (anti-H3K9ac and anti-H4Kac4) to detect the acetylated histones in the nuclei of different gametocyte stages. By means of these antibodies, we demonstrated that gametocyte treatment with TSA results in histone hyper-acetylation thereby leading to transcriptional activation. Histone hyper-acetylation following treatment of the malaria parasite with HDAC inhibitors have been earlier reported for *P. falciparum* blood stages (Andrews et al., 2008; Trenholme et al., 2014) and the asexual blood stages of *P. knowlesi* (Chua et al., 2017).

As a next step we aimed to investigate any deregulation of gene expression in immature, mature and activated gametocytes caused by TSA-treatment via microarray- and real-time RT-PCR-based analyses. Comparative transcriptomics between untreated and inhibitor-treated gametocytes identified 453 genes, which were more than 2-fold deregulated after 1 or 6 h following TSA-treatment. Among these 303 were more than 2-fold up-regulated, while 150 genes were down-regulated. Up-regulation of gene expression can be explained by increased euchromatin formation due to the loose contact between the negatively charged DNA and the acetylated histones, which hence counteracts gene silencing. Down-regulation of gene expression following TSA-treatment, on the other hand, which occurs in less than half of the identified genes, might be due to indirect effects. As mentioned earlier, PfHP1 was reported to bind to H3K9me3 to maintain the heterochromatin state, resulting in *ap2-g* repression (Brancucci et al., 2014), a process also involving PfHda2. It is postulated that the removal of acetyl groups by PfHda2 promotes histone methylation leading to PfHP1 binding (Coleman et al., 2014). Other studies have also reported the down-regulation of gene expression following treatment with HDAC inhibitors (Glaser et al., 2003; Chaal et al., 2010; Andrews et al., 2012b). Subsequent ChIP-qPCR analyses confirmed the general association of genes up-regulated in gametocytes following TSA-treatment with acetylated histones, particularly acetylated H3 and H4. The observed higher recovery rates achieved, when anti-H4Kac4 was used in the ChIP assays may be due to the facts that the antibody recognizes several lysine acetylation sites in H4 and that it additionally exhibits a minor binding to acetylated H2B.

An interesting finding in this study is the fact that the impairment of gene silencing during gametocytogenesis re-activates genes known to be crucial for blood stage replication. This was particularly observed for genes assigned to antigenic variation and cytoadherence and to RBC invasion by merozoites, or genes encoding exported proteins, which are particularly characteristic for the intraerythrocytic trophozoites. Interestingly, genes involved in antigenic variation and cytoadherence included ones coding for RIFINs and SURFINs, while only one out of 60 known *var* genes was identified. This might be explained by the fact that *var* gene activation and silencing is mostly accredited to the NAD-dependent histone deacetylases PfSir2A and PfSir2B (Duraisingh et al., 2005; Freitas-Junior et al., 2005; Tonkin et al., 2009) which are not affected following TSA-treatment. *Var* gene regulation is also linked to histone methylation, like H3K4me3-mediated *var* gene activation and H3K9me3-mediated *var* gene silencing (Chookajorn et al., 2007; Lopez-Rubio et al., 2007, 2009; Salcedo-Amaya et al., 2009), which further substantiate the reason why *var* genes were hardly identified. RIFINs are encoded by about 135 *rif* genes and comprise the largest family of antigenically variable molecules in *P. falciparum* (reviewed in Kirkman and Deitsch, 2014). H3K9 acetylation has been shown to control the expression of *rif* genes, which probably accounts for the reason why RIFINs were affected (Cabral et al., 2012). SURFINs are encoded by 10 surface-associated interspersed (*surf*) genes, which are located close or within the subtelomeric region of chromosomes. Little is known whether they are epigenetically regulated, but it is likely that they are regulated by histone PTMs (Mphande et al., 2008). Not identified by the microarray analyses were any of the genes coding for the 15 variant antigen-encoding *mc-2tm* genes. Furthermore, only one out of 35 STEVOR-encoding genes was up-regulated in gametocytes following TSA-treatment, suggesting that these gene families are not primarily affected in their expression levels by histone acetylation.

The majority of exported proteins regulated in their synthesis by histone acetylation include members of the PHIST (*Plasmodium* helical interspersed subtelomeric) family. The PHIST family comprises 89 proteins, the most of which have yet unknown functions. The PHIST protein family is characterized by a conserved domain of ~150 amino acids predicted to form four consecutive alpha helices and have been shown to be differentially expressed during the *Plasmodium* life-cycle (Warncke et al., 2016). The differential expression of these proteins may suggest an epigenetic mechanism. Invasion-related genes regulated in their expression by histone acetylation include previously identified surface proteins of merozoites, e.g., members of the MSP (merozoite surface protein) and the RON (Rhoptry neck) families (Proellocks et al., 2010; Beeson et al., 2016; Lin et al., 2016). Noteworthy, all of these identified gene families, i.e., *rif* and *stevor* and the ones encoding the PHIST family as well as the merozoite invasion-related families were reported to associate with heterochromatin markers (Flueck et al., 2009; Lopez-Rubio et al., 2009; Salcedo-Amaya et al., 2009) thereby maintaining default silencing of the majority of redundant members of multi-gene families (reviewed in Duffy et al., 2012).

While the above mentioned genes appear to be silenced by deacetylations of the associated histones after the parasites have entered the sexual pathways, other genes were apparently waiting to be activated by histone acetylation, once the parasite has been transmitted to the mosquito vector. Among others, these genes encode proteins important for midgut extravasation by the ookinete, like the secreted ookinete protein 25 (PSOP25), the secreted ookinete adhesive protein (SOAP), the von Willebrand factor A-domain related protein (WARP), the circumsporozoite and thrombospondin-related adhesion protein-related protein (CTRP), the cell traversal protein for ookinetes and sporozoites (CelTOS), or chitinase (CHT1) (reviewed in Pradel, 2007; Bennink et al., 2016).

To further justify our data, we selected one gene, *pfrnf1* (PF3D7_0314700), whose transcript was up-regulated following TSA-treatment of mature gametocytes, for further characterization. PfRNF1 possesses a C-terminal RING finger domain, which shows a high homology with the human E3 ubiquitin-protein ligase Praja-1. PfRNF1 has been annotated as a potential E3 ligase in *P. falciparum* as a component of the ubiquitin-mediated pathway (Ponts et al., 2008). TSA-treatment caused increased transcript and protein levels of PfRNF1 in mature gametocytes. Based on our data we postulate that under normal conditions deacetylation of histones H3 and H4 down-regulates PfRNF1 expression in mature gametocytes. The regulation of expression by histone deacetylases was recently also reported for the human RING finger domain protein RNF148 (Liu et al., 2013). We therefore suspect that PfRNF1 is a potential HDAC-regulated E3 ligase involved in ubiquitin-mediated pathways during gametocyte development, which might be important for regulatory processes during human-to-mosquito transmission of *Plasmodium*.

Our combined data highlight the role of histone acetylation in the control of gene expression during gametocyte development and transmission from the human to the mosquito, which may be exploited in malaria transmission-blocking strategies.

## Author contributions

Conceived and designed the experiments: CN, MK, ML, TV, and GP. Performed the experiments: CN, MK, OP, LO, and MF. Analyzed the data: CN, MK, OP, LO, AR, and GP. Wrote the manuscript: CN, MK, and GP. All authors read and approved the manuscript.

### Conflict of interest statement

The authors declare that the research was conducted in the absence of any commercial or financial relationships that could be construed as a potential conflict of interest.
