# Supplementary material for: Transcriptional Profiling Defines Histone Acetylation as a Regulator of Gene Expression during Human-to-Mosquito Transmission of the Malaria Parasite Plasmodium falciparum
Source: Front Cell Infect Microbiol. 2017 Jul 24;7:320. doi: 10.3389/fcimb.2017.00320 (PMC5522858; doi:10.3389/fcimb.2017.00320)
Supplement: Supplementary file 11 [file Table1.DOCX]

**Table S1: List of primers used in the study**

| PlasmoDB gene ID | Name/feature | Sense primer (5‘---3‘) | Anti-sense primer (5‘---3‘) | Position: |
| --- | --- | --- | --- | --- |
| qRT-PCR primers | | | |  |
| PF3D7_0113600 | SURF1.2 | ATTCCCATCTTGCTCAAACG | TGACCTTCTTGCACGACTTG |  |
| PF3D7_0113400 |  | CCCACGTTTTGCAACTAAAAA | TGCACTCTTCATTTGGGATG |  |
| PF3D7_0408700 | PLP1 | TCCCATCAATGCACAATGTC | GGCTGATCTCCACATTCCAT |  |
| PF3D7_1364900 | Ferrochelatase | GGAAGCCCAGAAAAACTAACC | GGAAGCCATAAAAACCTGTTCA |  |
| PF3D7_1477900 | ACS1b | TGGCCATTATTTCGATGGAT | CACGGAATCTTTTCACCTTCA |  |
| PF3D7_1413600 |  | TTCTATCGCCCAACTCATGT | GATTGGTGGACATTATTTCCTG |  |
| PF3D7_0207300 |  | ATTGTTGGGTTTTTGCTTCG | CAGGACATTCCGATCCTGTT |  |
| PF3D7_0911300 | CRMP1 | ACCCGTACTTGGAACGTGTG | TCCACATTGAACAGGAAGCA |  |
| PF3D7_1334400 | MSRP4 | ATCCCCAGTACAAACCACCA | TGAAATTGTCAAACCTGCTACG |  |
| PF3D7_1016500 |  | CGAATTTGAACTCTGATTTGGA | CCACATGCCATTTCATCTACA |  |
| PF3D7_1147000 | LARP | GCACCCATTTTACAACCGATA | GTCCATTTGATGGGTTGGTT |  |
| PF3D7_0937000 |  | TCTTGGCGAATACTCTGATGG | TGCTTCCCACCTTTACAACA |  |
| PF3D7_0315900 |  | AAGCATATTTGCAAACAACGAA | CGATTCACTTAAGCAGTTCAA |  |
| PF3D7_0318800 | Triosephosphate isomerase, putative | CCGTATTCCAAAAATCAACCA | TCACTCGAATTATTCCGTTCG |  |
| PF3D7_0620200 |  | TGCCCTTAAGAAAACCAAAGAA | GATGAACATCCATCAGCTTCA |  |
| PF3D7_0404100 | ApiAP2 | AGGGGGAACATAAGGATGGT | CCAAACTTGCACACAGGAAA |  |
| PF3D7_1427600 | MIT3 | GGGAAAAATCAGATCCTACTCCA | CATCATCCAAGGATTGATCTGT |  |
| PF3D7_1230300 | SPM2 | AGAAAACCAGGGTGTTCCAA | TGAAGGTAGCTTGGGCTTTG |  |
| PF3D7_0113300 |  | TCAAACGAAGCAACACATCA | TGGCGCTCTAAAAAGTTGGT |  |
| PF3D7_1038600 |  | TCGGACTAACAACGGTAGCA | TGTGAGGGGAGAAATACTGCTT |  |
| PF3D7_0408600 | SIAP1 | CCACATGGTGGAAAACACAG | CCACCCATAGAACCTTTTGG |  |
| PF3D7_0314700 | PfRNF1 | AACGCAAATGTTGATTGCTG | AGGCTTCTGTGCGATCTTGT |  |
| PF3D7_1133400 | AMA1 | GGATTATGGGTCGATGGA | GATCATACTAGCGTTCTT |  |
| PF3D7_1455800 | PfCCp2 | TCGGATGGAGAATCCGTT | GTATCCCATGTCTTGTGA |  |
| PF3D7_0717700 | Seryl tRNA ligase | AAGTAGCAGGTCATCGTGGTT | TTCGGCACATTCTTCCATAA |  |
| Primers used for ChIP | | | |  |
| PF3D7_1218600 | arginine-tRNA ligase | AGCTAAAGAGATGCATGTTGGTCATT | GAGTACCCCAATCACCTACATGA | coding region |
| PF3D7_0717700 | Seryl tRNA ligase | AAGTAGCAGGTCATCGTGGTT | TTCGGCACATTCTTCCATAA | coding region |
| PF3D7_1222600 | AP2-G | TGGTGGTAATAAGAACAACAGAGGT | CCATCATAATCTTCTTCTTCGTCG | coding region |
| PF3D7_0426000 | PfEMP1 | TGACGACTCCTCAGACGAAG | CTCCACTGACGGATCTGTTG | coding region |
| PF3D7_0314700 | PfRNF1 | TGTATCGTGACAAGGGTCCA | TCTTGTTTGAAGTGGGTTCGT | coding region |
| PF3D7_0314700 | PfRNF1 | TGACATTTTCTTCTGTAACGGCA | GAGGCCCAAGAAACACCAGT | promotor region |
| PF3D7_1428400 | WD and tetratricopeptide repeats protein 1, putative | ACTGCATGGGAAATGAAGGA | ACCTCGCCATTATCAGAAGCA | coding region |
| PF3D7_1428400 | WD and tetratricopeptide repeats protein 1, putative | AGCACCATCAGAAATGATCCA | AGCTTATGATGCTTCATCGATGTTT | promotor region |
| PF3D7_0219800 | Plasmodium exported protein (PHISTc), unknown function | AGCACTTATGAAACGAACCCA | CTCGAGGGACCCCTTTTTCA | coding region |
| PF3D7_0219800 | Plasmodium exported protein (PHISTc), unknown function | ACTTTTGGACACTTTTATCAAAGGATTA | TGTACACAGGCCATACCTGA | promotor region |
| PF3D7_0620200 |  | TGCCCTTAAGAAAACCAAAGAA | GATGAACATCCATCAGCTTCA | coding region |
| PF3D7_0620200 |  | cccctcaggcaacaaaaata | tctgcacgattaaagacgaaaa | promotor region |
| PF3D7_0926600 |  | AACTTCTATCCAAGGGGGCA | TCTCCCCCTCACAGGTATCTC | coding region |
| PF3D7_0926600 |  | TAATGATTTACCTCTTCAATGGTGC | TATGTGCATCATACAAAACGGAGC | promotor region |
| PF3D7_0821500 | ribosomal RNA small subunit methyl-transferase NEP1, putative | TCGGATGACCCAAATGATTAC | TTTTTCTTCCTTTGTTCATCATC | coding region |
| PF3D7_0821500 | ribosomal RNA small subunit methyl-transferase NEP1, putative | catcgtcttgtttgttctcca | ccacagttaaccatcgctagg | promotor region |
| Primers used for recombinant protein expression | | | |  |
| PF3D7_0314700 | PfRNF1 | aGatCtGCGGCCGCCAAACGCATGCAAAAGAA | aGatCtCTGCAGAGGCTTCTGTGCGATCTTG |  |
| Primers used for generation of HA-strep tagged parasite lines | | | | |
| PF3D7_0314700 | PfRNF1 | actagtcctccCCGCGGGTTTCCCAGAAGAATGGCAAG | ctcctttactCTCGAGCTTCTTATAACTATTTCGAAG |  |
| Primers used to confirm PfRNF1-HA-Strep tagging | | | | |
| PF3D7_0314700 | 1 and 2 | CACGTATGGATAATCGCCTTG | TCAAAATAAATTGACGCGATTAAA |  |
|  | 3 and 4 | AGATAAAATTTGTAGAGA | CAGCGGCATAATCTGGAA |  |
